# Immunodeficiency and CARD-BCL10-MALT1

**DOI:** 10.18632/oncotarget.4467

**Published:** 2015-06-14

**Authors:** Rebeca Pérez de Diego

**Affiliations:** Immunogenetics of Diseases Laboratory, Innate Immunity Group, IdiPAZ Institute for Health Research, La Paz University Hospital, Madrid, Spain

**Keywords:** primary immunodeficiency, BCL10, CID, CBM complex

Three members of the caspase recruitment domain (CARD) family of adaptors can form heterotrimers with B-cell lymphoma 10 (BCL10) and mucosa-associated lymphoid tissue lymphoma translocation gene 1 (MALT1); they form the cytosolic complex CARD-BCL10-MALT1 (CBM). Three CARD-family adaptor proteins have been shown to form CBM complexes: CARD9, CARD10 (also known as CARD-containing MAGUK protein 3 [CARMA3]), and CARD11 (also known as CARMA1). The CBM complex mediates NF-κB and mitogen-activated protein kinase (MAPK) activation in a cell-type-specific and non-redundant manner after the stimulation of various immune receptors. The NF-κB transcription factor plays a critical role in innate and adaptive immune regulation, cell memory, cell survival/apoptosis, and cell cycle progression. Various cell types contain members of the CARD family: CARD9 in myeloid cells [1], CARD10 in epithelial cells [2, 3], and CARD11 in lymphocytes [1]. The CBM complex acts downstream from the following receptors: the T-cell (TCR) and B-cell (BCR); BAFFR; TLR4; several natural killer receptors (NKRs), such as NK1.1, Ly49H, NKG2D, and Ly49D; C-type lectin receptors, such as dectin-1, dectin-2, or Mincle; FcγR; and the G protein–coupled receptor (GPCR). The first studies on CBM complex proteins were focused on MALT1. In 1983, MALT1 was discovered to be implicated in a pathology associated with MALT lymphoma, a B-cell lymphoma frequently involving the gastrointestinal tract. This type of tumour constitutes the most common subset of extranodal non-Hodgkin's lymphomas [4]. In 1999, chromosomal translocations of *BCL10* were also shown to be associated with MALT lymphomas [5].

We have recently reported autosomal-recessive, human BCL10 deficiency in a child with a combined immunodeficiency [6]. The BCL10 mutation is a loss-of-expression, loss-of-function mutation that reveals various degrees of BCL10 dependence for several innate and adaptive signalling pathways. Human BCL10 deficiency shows minimal differences in the bcl10^−/−^ mouse phenotype. However, there are significant differences in the phenotypes with CARD9, CARD11, and MALT1 human deficiencies, which redefines the role of BCL10 in the human immune system as shown to date. At a clinical level, BCL10 is a new deficiency for patients with a combined immunodeficiency, which rather than being a leaky phenotype is instead susceptible to severe progression. The BCL10 deficiency had a homozygous splice-site mutation (G/A) affecting the first G residue of intron 1 (g.85741978C > T; IVS1+1G>A). This nucleotide is the first nucleotide of intron 1 and is invariant, given it is part of the donor site for splicing. The *BCL10* mutation resulted in an absence of wild-type mRNA and protein. This patient was found to have a profound deficit of memory T and B cells and abnormally low numbers of CD4^+^ central memory T cells and CD4^+^ regulatory T cells. T-cell proliferation was blocked following TCR stimulation *in vitro*. Most B cells (97.1%) were naïve, which is consistent with the profound hypogammaglobulinemia observed in this patient [6]. Human BCL10 deficiency affected the final maturation of the T and B cells into memory cells, but not the development of naïve T and B cells. Myeloid cells (which form CARD9-BCL10-MALT1 heterotrimers) displayed a normal pattern of cytokine production in response to stimulation with TLR1/2, TLR4, TLR2/6, and Dectin-1. In contrast, BCL10-deficient fibroblasts (in which the heterotrimer formed is CARD10-BCL10-MALT1) were unable to translocate the NF-κB p65 subunit to the nucleus, and cytokine production in response to stimulation with TLR4, TLR2/6, and Dectin-1 was abolished.

The human inherited defect of BCL10 described in Torres, et al. is added to other mutations described in MALT1 and two CARD proteins (9 and 11) [7]. Interestingly, these primary immunodeficiencies underlie various immunological and clinical phenotypes, ranging from invasive fungal infections in CARD9 deficiency to severe forms of combined immunodeficiency in the CARD11, MALT1, and BCL10 deficiencies, with various lymphocyte subpopulations and immunoglobulin levels [7]. The BCL10-deficient patient and two of the four MALT1-deficient patients died, whereas one MALT1-deficient patient and one of the two CARD11-deficient patients have undergone bone marrow transplantation. The other CARD11-deficient patient is awaiting transplantation. Bone marrow transplantation appears to be the best treatment for these disorders, given the tragic outcomes of the patients not treated in this manner. However, in CARD9 deficiency, innate immunity is affected in a very specific way, as shown by the patterns of susceptibility to invasive disease, which are typically caused by a single fungal pathogen in each patient. In these patients, adjuvant granulocyte-macrophage colony-stimulating factor (GM-CSF) treatment appears to have been beneficial. In addition, for CARD9-deficient patients with extensive dermatophytosis, terbinafine has been shown to be successful as a first-line treatment and posaconazole as a second-line treatment. Finally, taking into account that the overproduction of MALT1 and BCL10 has been implicated in MALT lymphoma, and several therapeutic studies have thus focused on inhibiting expression of the CBM complex, it is important to consider that BCL10 deficiency appears to have an impact on lymphocytes and fibroblasts but not on myeloid cells. This finding is highly relevant and should be taken into account when considering treatment options for MALT lymphoma because BCL10 inhibition can have consequences for lymphocytes and non-hematopoietic cells. These new primary immunodeficiencies highlight the relative importance of the mutated components of human CBM complexes in immunity.
